# Nonlinearity- and dispersion- less integrated optical time magnifier based on a high-Q SiN microring resonator

**DOI:** 10.1038/s41598-019-50691-2

**Published:** 2019-10-03

**Authors:** Arijit Misra, Stefan Preußler, Linjie Zhou, Thomas Schneider

**Affiliations:** 1 THz-Photonics Group, Technische Universität Braunschweig, Schleinitzstraße 22, 38106 Braunschweig, Germany; 2 State Key Laboratory of Advanced Optical Communication Systems and Networks, Shanghai Jiao Tong University, 200240 Shanghai, China

**Keywords:** Integrated optics, Microwave photonics

## Abstract

The ability to measure optical signals with fast dynamics is of significant interest in many application fields. Usually, single-shot measurements of non-periodic signals can be enabled by time magnification methods. Like an optical lens in the spatial domain, a time magnifier, or a time lens, stretches a signal in the time domain. This stretched signal can then be further processed with low bandwidth photonics and electronics. For a robust and cost-effective measurement device, integrated solutions would be especially advantageous. Conventional time lenses require dispersion and nonlinear optical effects. Integration of a strong dispersion and nonlinearities is not straightforward on a silicon photonics platform and they might lead to signal distortions. Here we present a time magnifier based on an integrated silicon nitride microring resonator and frequency-time coherence optical sampling, which requires neither a dispersion, nor a nonlinearity. Sampling of signals with up to 100 GHz bandwidth with a stretching factor of more than 100 is achieved using low bandwidth measurement equipment. Nevertheless, with already demonstrated integrated 100 GHz modulators, the method enables the measurement of signals with bandwidths of up to 400 GHz. Since amplitude and phase can be sampled, a combination with the spectrum slicing method might enable integrated, cost-effective, small-footprint analog-to-digital converters, and measurement devices for the characterization of single irregular optical signals with fast dynamics and bandwidths in the THz range.

## Introduction

Observation of the temporal shape of a short-lived optical signal is important for many different applications in the fields of optical physics, communication technology, and spectroscopy. Amplitude and phase of short optical signals can be measured in the frequency and time domain by nonlinear methods like frequency-resolved optical gating (FROG)^1^. Conventionally, single-shot measurement methods like FROG require significant pulse energies^1–3^ while, in application fields like optical communications and radar, usually signals with a very low signal-to-noise ratio are incorporated. Although very efficient high-order digital modulation formats are used today, the optical communication bandwidth is ever increasing towards even terahertz range^4–6^. Continuing advancements in the complementary metal oxide semiconductor (CMOS) technology have led to astonishingly fast digital signal processors. But, the analog bandwidth of wideband digitizers or so-called analog-to-digital converters (ADCs) is still one of the major bottlenecks^7^. By spectral slicing^8^, i.e. the parallel coherent detection of low bandwidth spectral slices of a broadband signal, high bandwidth arbitrary input signals can be measured. However, it requires a narrow-spacing, flat-top wavelength demultiplexer to slice the broadband spectrum before homodyne detection and digitization with low-speed electronics is possible.

Therefore, a system capable of stretching a short optical signal temporally to a scale that enables the measurement with conventional low-bandwidth electronic systems is essential for present day applications. Usually, a time-lens is utilised for the temporal magnification of short input signals. Like a lens in the spatial domain, a time lens can magnify ultrafast time evolutions in time^9–15^. In general, there exists a mathematical equivalence between spatial diffraction and time domain dispersion. Moreover, temporal quadratic phase modulation is analogous to spatial phase modulation by a conventional lens. Therefore, a suitable combination of dispersion and quadratic phase modulation can result in a system where the temporal behavior of a signal can be scaled in an arbitrary way^11,12,15^.

The concept of time magnification was first proposed in the electrical domain^9^ before it was implemented in optics. A typical optical time magnifier system can be realized either by exploiting time-frequency conversion^14,16^ analogous to the Fourier transform properties of a spatial lens^17^, by using an electro-optic phase modulator^18^ or by utilizing parametric nonlinear wave mixing processes like sum-difference frequency generation^11^ and four-wave mixing (FWM)^13,19,20^. A method which can map the incoming signal spectrum to a temporal waveform is the dispersive Fourier transform (DFT)^21^. DFT has been instrumental for photonic time stretched analog to digital converters (TSADC) for fast digitization of high bandwidth signals^22,23^. A high bandwidth data signal is modulated on a chirped pulse by an electro-optic intensity modulator. This step acts as a time-to-frequency conversion for the input signal. A dispersive unit afterwards stretches the signal in time before detection by a conventional photodiode^22^. A combination of DFT and time lens has been used for the single shot complete transient characterization, where the spectral and temporal behavior of the signal is retrieved separately followed by a Gerchberg–Saxton algorithm to get the complete intensity and phase information^24^.

Another method for photonic analog to digital conversion is based on a fiber loop with optical amplification to generate copies of the input signal in combination with dispersion compensated fiber, large effective-area fiber, and highly nonlinear fiber in order to magnify successive parts of each copy using dispersion engineering and FWM^20^.

Most of the aforementioned time magnification methods require a strong dispersion or nonlinearity. Integrated, small footprint, and economical measurement devices would be advantageous for various application fields. However, the on chip integration of nonlinear optical effects or elements with a strong dispersion is challenging using conventional commercial fabrication facilities. Furthermore, nonlinearities require high signal intensities and long interaction lengths, and higher-order dispersion and other nonlinearities can lead to a distortion of the signal.

Here a time-magnifier – based on the multiplication of the signal spectrum with a frequency comb, enabled by an integrated silicon nitride microring resonator, and the following convolution with a rectangular frequency comb by two cascaded intensity modulators – which neither requires a dispersion nor a nonlinearity will be presented. Thus, full integration into a photonic integrated circuit is straightforward. Additionally, the measurement device can achieve sampling rates of up to 4 times the RF bandwidth of the integrated modulator with the highest bandwidth. If combined with spectrum slicing and co-integrated electronics, single, irregular signals with THz bandwidths could be characterized by low bandwidth photonics and electronics in a single integrated device.

## Results

### Theory

**Figure 1:**
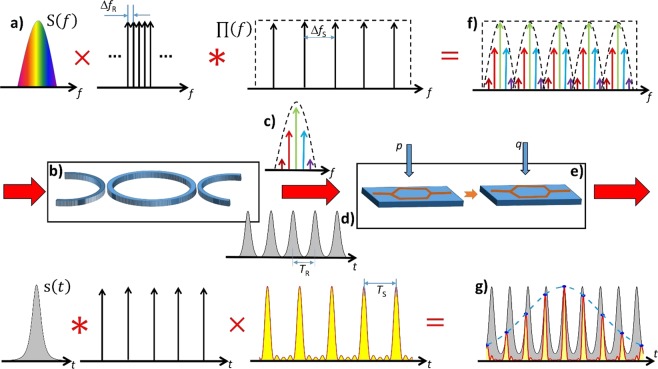
Basic concept of the proposed time magnifier in the frequency (top) and time domain (bottom). The multiplication between the optical input signal spectrum (**a**) with a frequency comb realized by the transfer function of a ring resonator with an infinitely high finesse (**b**) results in a convolution between the input signal and a Dirac delta comb in the time domain, or an infinite number of time copies of the input signal (**d**). In the next step, a set of two cascaded modulators (**e**) enables the convolution in the frequency domain between the discrete signal spectrum (**c**), produced by the ring resonator and a rectangular frequency comb. In the corresponding time domain, this is the multiplication of the signal copies with a sinc pulse sequence (**g**). Since the free spectral range of the ring is slightly different from the repetition rate of the sequence, the signal copies are sampled at different positions. The sampling points are the integral of the sequence over the repetition rate (blue dots) and the envelope of the sampling points gives the stretched input pulse (blue dashed line).

The basic working principle of the dispersion- and nonlinearity-less time magnifier is very simple. An integrated ring resonator with a round trip time, much longer than the duration of the single isolated input signal, is used to sample the input signal in the frequency domain (Fig. 1c), i.e. to generate several copies of the input signal in the time domain (Fig. 1d) with a distinct repetition rate. In a second step, two cascaded intensity modulators are used to sample the different copies in the time domain by using the frequency-time coherence method^25^. Due to a subtle difference between repetition and sampling rate, the sampled values are taken from different positions of the signal copies (Fig. 1g). The envelope over these sampling values is the time-stretched and sampled input signal (dashed line in Fig. 1g).

For the explanation of the ring resonator and the sampling stage acting together, a comprehensive formulation of the interaction in the time and equivalent frequency domain offers a better description. The optical powers required for the experiment are far too low to generate any nonlinear optical effects and dispersion induced signal broadening was absent in our experimental outcome. Therefore, these effects have been neglected for the sake of simplicity in the theoretical formulation.

An arbitrary signal in the frequency (top) and time domain (bottom) is depicted in Fig. 1. They are connected via the frequency-time coherence described by the well-known transform equations of Jean Baptiste Joseph Fourier^26^. Assume that the input signal *s*(*t*) in time and *S*(*f*) in the frequency domain, has a higher optical bandwidth *B*_*o*_ than the free spectral range (Δ*f*_*R*_) of the ring (Fig. 1a). Additionally, for the sake of simplicity, consider that the finesse (Δ*f*_*R*_/Δ*f*), with Δ*f* as the full-width at half-maximum is unlimited, i.e. the ideal transmission spectrum at the drop port of the ring can be seen as an infinite number of discrete Dirac delta frequency lines equally separated by Δ*f*_*R*_. Thus, the ring resonator multiplies the signal spectrum with a Dirac frequency comb, i.e. it extracts single, equidistant frequency lines, leading to a discrete signal spectrum (Fig. 1c). This process can be considered as sampling in the frequency domain. With the definition of Dirac delta sequences in frequency and time domain as:

1 $${\rm{III}}(x,Y)=\mathop{\sum }\limits_{n=-\infty }^{\infty }\delta (x-nY),$$

with *x* = *f*, *t*, the output of the ring resonator can be expressed in frequency and time domain as:

2 $${S}_{RR}(f)=S(f)\times {\rm{I}}{\rm{I}}{\rm{I}}(f,\Delta {f}_{R}){\rm{.}}$$

3 $${s}_{RR}(t)=\frac{1}{\Delta {f}_{R}}\mathop{\sum }\limits_{n=-\infty }^{\infty }s(t-\frac{n}{\Delta {f}_{R}})$$

Consequently, the sampling in frequency domain (Eq. (2)), carried out by the ring resonator, leads to an infinite number of equal copies of the input signal in the time domain as expressed by Eq. (3) ^27–29^. If the finesse is considered to be finite, an additional decaying factor has to be included in Eq. (2). However, since the method uses an electronic post-processing, as will be described later, this decay plays no role for the concept and will be neglected here.

In a second step, the discrete signal spectrum (Fig. 1c) is convolved with a rectangular frequency comb, leading to equal copies of the discrete signal spectrum in the frequency domain (Eq. (4), Fig. 1f),

4 $${S}_{s}(f)={S}_{RR}(f)\,\ast \,[{\rm{I}}{\rm{I}}{\rm{I}}(f,\Delta {f}_{s})\times {\prod }^{}(f)].$$

Where ∏(*f*) is a rectangular function defined around the optical carrier frequency *f*_c_. For a frequency comb with an odd number of lines, it can be written as:

5 $$\prod (f)=\{\begin{array}{ll}1 & -\frac{N\Delta {f}_{s}}{2}\le {f}_{c}\le \frac{N\Delta {f}_{s}}{2}.\\ 0 & elsewhere\end{array}$$

In the equivalent time domain, Eq. (4) can be expressed as:

6 $${s}_{s}(t)=\frac{1}{\Delta {f}_{R}}\mathop{\sum }\limits_{n=-\infty }^{\infty }s(t-\frac{n}{\Delta {f}_{R}})\times [{\rm{I}}{\rm{I}}{\rm{I}}(t,\frac{1}{\Delta {f}_{s}})\,\ast \,N\mathrm{sinc}(N\Delta {f}_{s}t)],$$

where ${\rm{sinc}}(t)=\,\sin (\pi t)/(\pi t)$ is the sinus cardinalis or sinc function. For a time shift of *τ* = 1/(*N*Δ*f*_s_) the sinc function is orthogonal to a time shifted version of itself. Hence, the convolution between the Dirac delta sequence and the sinc function in the right brackets of Eq. (6) gives the sinc pulse sequence as^30–32^

7 $${\rm{I}}{\rm{I}}{\rm{I}}(t,\frac{1}{\Delta {f}_{s}})\,\ast \,N\mathrm{sinc}(N\Delta {f}_{s}t)=\mathop{\sum }\limits_{k=-\infty }^{\infty }N{\rm{sinc}}(N\Delta {f}_{s}(t-\frac{k}{\Delta {f}_{s}}))=\frac{\sin (\pi N\Delta {f}_{{\rm{s}}}t)}{\sin (\pi \Delta {f}_{{\rm{s}}}t)\,}.\,$$

The bandwidth and duration (from the peak to the first zero crossing) of the single pulses in the sequence is given by, *B* = *N*Δ*f*_s_. The process carried out in the two coupled intensity modulators can be seen as a sampling in the time domain. The sampled signal can be expressed as:

8 $${s}_{s}(t)=\frac{1}{\Delta {f}_{R}}\mathop{\sum }\limits_{n=-\infty }^{\infty }s(t-\frac{n}{\Delta {f}_{R}})\times \mathop{\sum }\limits_{k=-\infty }^{\infty }N{\rm{sinc}}(N\Delta {f}_{s}(t-\frac{k}{\Delta {f}_{s}})).$$

Following Eq. (8), the ring resonator generates equal time copies of the input signal with a repetition time of ${T}_{R}=1/\Delta {f}_{R}$ and in the following step, these time copies are multiplied with an infinite sequence of sinc pulses with a repetition time of ${T}_{s}=1/\Delta {f}_{s}$. If ${T}_{s}\approx m{T}_{R}$, with *m* = 1,2,3, …, the stretching factor of the input signal is given by:

9 $$\Delta S=\frac{{T}_{seff}}{|{T}_{seff}-m{T}_{R}|}=\frac{\Delta {f}_{R}}{|\Delta {f}_{R}-m\Delta {f}_{seff}|}$$

Here *T*_*seff*_ and $\Delta {f}_{seff}$ are the effective repetition time and rate respectively. If not every, but every second or third copy will be multiplied by the sampling pulses, the repetition time has to be multiplied by 1/2 or 1/3 to get the effective values. For a practical system, the number of useful time copies is restricted by the optical input power as well as the finesse and losses of the ring resonator. Therefore, the maximum stretching factor can be achieved for *m* = 1.

If the envelope is compressed by the stretching factor, it gives the sampling of the original pulse. Henceforth, this will be called compressed sampling rate $\Delta {f}_{CS}=\Delta {f}_{seff}\Delta S$. Furthermore, it will be shown in the discussion section that the achievable maximum effective compressed sampling rate can be defined by the bandwidth of the rectangular frequency comb as $\Delta {f}_{CSeff}=B=N\Delta {f}_{s}$.

The convolution of the discrete signal spectrum with a rectangular frequency comb (Eq. (4)) is carried out by two cascaded intensity modulators, where each of them is driven with one or more synchronized radio frequencies (RF) (Fig. 1e). To achieve such a frequency domain convolution, both modulators have to be adjusted in a way, that the generated spectral copies have the same amplitude and phase^30^. For the sake of simplicity, it is assumed that just one RF frequency is applied to each modulator. The first modulator is driven with the frequency Δ*f*_*s*_. Thus, it generates three equal copies of the discrete signal spectrum, i.e. the central frequency of the carrier at *f*_*c*_ and two sidebands at ${f}_{c}\pm \Delta {f}_{s}$. The second modulator is then driven with a frequency 3Δ*f*_*s*_. Again, the second modulator produces three equal copies of its input spectrum. Hence, the second modulator enhances the number of equal spectral copies to *N* = 9 (see Fig. 1f). According to Eq. (8), this corresponds to a multiplication of the time copies with a sinc pulse sequence with eight zero crossings in the equivalent time domain (Fig. 1g). If the first modulator is driven with *p* and the second with *q* RF frequencies, the number of spectral copies can be enhanced to $N=(2p+1)(2q+1)$ with the achievable pulse bandwidth of $B=N\Delta {f}_{s}$.

The sampled values are the integral of the sinc pulse sequence over its repetition rate, which is again Δ*f*_*s*_ and if a coherent receiver is used, the full field can be sampled^33^. Consequently, the amplitude and phase values can be extracted by low bandwidth photodiodes and electronics. If a frequency tripler is used to generate the input frequency for the second modulator, the maximum required electronic bandwidth and the bandwidth of the photodiode is Δ*f*_*s*_. So, even with low RF bandwidth electronics and photonics, high bandwidth signals can be measured.

### Proof-of-concept experiment

**Figure 2:**
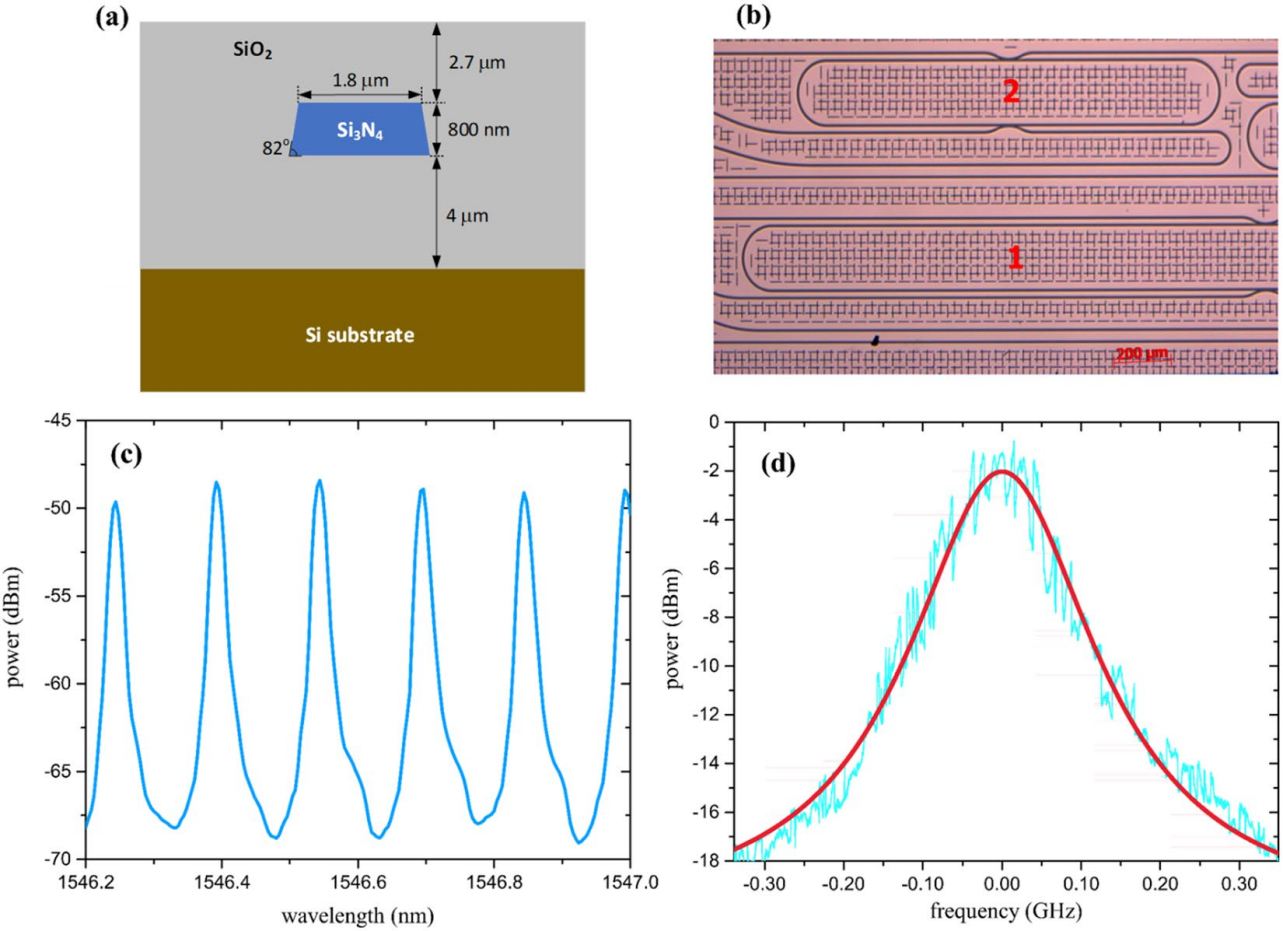
Characteristics of the ring resonator. (**a**) Cross-section of the waveguide. (**b**) Top layout of a portion of the chip. For the experiment, ring 1 with a free spectral range of 18.774 GHz has been used. Due to lack of space, only half of the ring 1 is shown. However, the layout is the same as that of ring 2. (**c**) Transfer characteristic at the drop port measured using a broadband source and an optical spectrum analyser with 0.02 nm resolution. (**d**) Single resonance peak at 1545.94 nm, measured by an optical power meter and a frequency scan (cyan) with a Mach-Zehnder modulator driven with a tunable RF frequency. The red curve corresponds to the Lorentzian fit to the measured data. The calculated finesse is 145.

To proof the theoretical predictions, an integrated silicon nitride microring resonator has been used to create time domain replicas of the input signal and two cascaded conventional LiNbO_3_ Mach-Zehnder modulators have been used for the convolution with a rectangular frequency comb. A cross sectional and top view of the fabricated ring can be seen in Fig. 2a,b, respectively. Details of the fabrication process can be found in the methods section. For the experiments, we have used the ring with the lowest free spectral range of $\Delta {f}_{R}=18.774$ GHz (labeled with 1 in Fig. 2b). The transfer function of the rings drop port, measured with a broadband light source and an optical spectrum analyzer (OSA) can be seen in Fig. 2c. Since the resolution of the spectral measurement is restricted by the OSA (2.5 GHz @ 1550 nm), we have scanned one of the frequency resonances by a sideband generated with a Mach-Zehnder modulator, driven with a tunable radio frequency generator. From this measurement the full width at half maximum (FWHM) of the resonance was found to be Δ*f* = 128.9 MHz (Fig. 2d), leading to a finesse of around 145 and a Q-factor of ≈ 1.5 × 10^6^.

**Figure 3:**
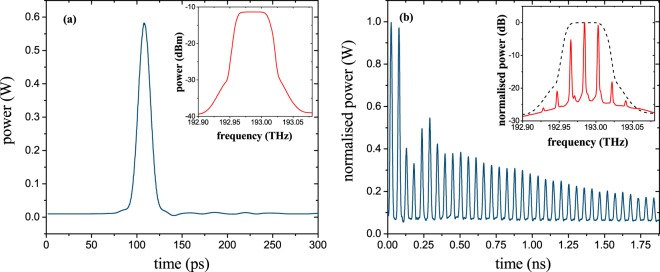
Time and frequency domain (insets) representation of the signal at the input (**a**) and output of the ring (**b**). The input signal (**a**) is a pulse train with a repetition rate of 100 MHz (10 ns), produced by an Erbium-doped, mode-locked fibre laser and bandwidth limited to 60 GHz by a waveshaper (inset in (**a**)). Frequency lines are carved out from the signal spectrum by the transfer function of the ring (inset in (**b**)). Since the finesse of the ring is finite and due to losses, the ring produces copies for around 2 ns. The amplitude behavior of the copies after the ring is defined by the ring parameters and the input power but not by the input signal. Hence, it can be equalized by post-processing of the data. Please note from the inset in (**b**) that new frequency lines outside the original spectrum (dashed line) cannot be seen. Thus, the ring does not lead to any visible nonlinear optical effects.

Since no single, isolated broad bandwidth signal source was available for the experiment, the test signal was generated with a passively mode-locked femtosecond Er-doped fiber laser (Toptica FemtoFErb1560). In contrast to the simplified theory, the finesse of the ring resonator is finite and there is attenuation in the ring. These lead to vanishing of the copies long before the next input pulse enters the ring. Consequently, there is no interaction between residual optical power from the copies and the power of the next input pulse. As can be seen from Fig. 3b, the useful copies last for a duration of about 2 ns. The repetition time of the mode-locked laser was 10 ns (100 MHz) and thus five times longer than the duration of the copies. So, although a periodical signal has been used for the proof of concept experiment, the time magnifier works on each single pulse. The bandwidth of the generated laser pulses was 30 THz and by far too broad for the electronic measurement devices available in the lab to verify the proposed method. Thus for the proof of concept experiments, the signal bandwidth was reduced to 60 and 100 GHz by a waveshaper. This can be seen as a sliced part of the broader spectrum. For a measurement device based on spectral slicing, a number of these spectral slices would be processed in parallel. The reduced bandwidth of the mode-locked frequencies results in pulses with an FWHM duration of around 15.55 ps and 10.37 ps, respectively. The higher the bandwidth of the sliced part of the spectrum, the lower is the number of required parallel branches. As will be discussed later, the maximum bandwidth of the pulses which can be measured is restricted to four times the bandwidth of the incorporated Mach-Zehnder modulator with the highest RF bandwidth. Since one 40 GHz modulator has been used, this would correspond to only 160 GHz per slice. However, with 100 GHz modulators, already demonstrated on a chip^34^, a bandwidth of up to 400 GHz per spectral slice would be feasible.

The time and frequency representation of the single pulses before the ring can be seen in Fig. 3a. These pulses were coupled into the microring resonator. Due to the drop port transfer characteristic of the ring, single frequencies were extracted from its spectrum (inset in Fig. 3b). According to Eqs (2) and (3), these single frequency lines correspond to a number of copies of the input pulse in the time-domain (Fig. 3b). As can be seen from the inset in Fig. 3b, there are no newly generated frequency lines outside the original pulse spectrum. According to our measurements, an optical input power of 25 dBm was necessary to generate small sidebands by four wave mixing in the used ring and an input power of as much as 34 dBm is required to generate a frequency comb. Therefore, with an optical input power of just 5 dBm at the input bus waveguide, as used for the experiments, the nonlinearity of the ring resonator and possible accompanied distortions are negligible.

Due to the roundtrip loss in the ring, the temporal replicas are decreasing. In Fig. 3b, the third and fourth copy have much lower power than expected and the power of other replicas slightly fluctuates. This is the same for all signals and independent of the input power. This behavior arises from spurious resonances with much lower power than the principal resonance caused by (i) sidewall corrugation of the fabricated ring^35^ and (ii) presence of coupling sections which act as perturbation for ring resonator^36^. More comprehensive explanation on this phenomenon can be found in^37^. Since this effect depends on the fabricated ring and not on the input signal, exactly the same fluctuation can be seen for all input signals and it can be compensated by post-processing of the data. Therefore, it does not influence the measurement. Additionally, an integrated photodiode at the through port of the ring with a bandwidth comparable to the ring FSR can be used to get the envelope of the power distribution as well as the repetition rate of the copies for post processing. By monitoring the actual repetition rate, temperature stabilization of the ring can be avoided.

As depicted in Fig. 1, the time copies generated by the ring resonator were fed into two cascaded modulators. The first modulator was driven with a frequency Δ*f*_*s*_ of 12 GHz, generated by a phase-locked loop along with a voltage controlled oscillator (PLL, see methods section). The bias voltage and RF power of the modulator was adjusted in a way, that the upper and lower copies of the input spectrum had the same amplitude and phase as the spectrum in the middle^25,31^. The second modulator was driven with three times the radio frequency Δ*f*_*s*_, generated by a frequency tripler. As discussed in the theory section, the second modulator enhances the number of spectral copies to nine. This corresponds to a convolution of the input spectrum with a nine-line rectangular frequency comb. In the time domain, it is the multiplication of the time-copies with a sinc pulse sequence with eight zero crossings^25,31^ (Eq. (8)).

**Figure 4:**
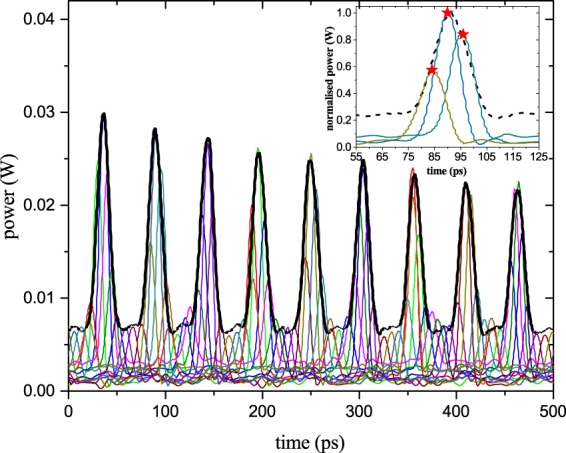
Sampling of the signal replicas appearing at the output of the ring resonator. The cascaded modulators convolve the discrete input spectrum with a nine-line rectangular frequency comb with a frequency spacing of Δ*f*_*s*_ = 12 GHz. In the corresponding time domain, this results in a multiplication of the signal copies with a sinc pulse sequence. The sequence can be shifted through the signal to sample by a phase shift of the electrical signal used to produce the frequency comb. Each colour represents one sinc pulse sequence with a different RF phase. Regardless of the amplitude difference or pedestal of the pulses, the sampling follows the signal. In the inset, the sampling of just one copy with three different sequences is shown. The stars represent the sampling points achieved by an integration of the sequences over its repetition time. The sampling pulses are distorted since their bandwidth was 108 GHz and thus broader than the bandwidth of the photodiode (100 GHz). However, this does not influence the result.

By changing the phase of the radio frequency, generated by the PLL, the sinc pulses can be shifted through the replicas as shown in Fig. 4. Each color represents a measurement with a sinc pulse sequence with a different phase. As can be seen, the sampling follows the signal regardless of the amplitude or an additional pedestal. The inset in Fig. 4 shows a zoom into one of the copies. The stars represent the sampling points achieved by an integration of the sinc pulses over the sampling time (repetition time of the sinc pulses). The bandwidth of the sampling pulses is $B=N\Delta {f}_{S}=9\times 12\,{\rm{GHz}}=108$ GHz and thus higher than the bandwidth of the used photodiode (100 GSa/s) as well as the electrical oscilloscope (70 GHz). As a result, the measured sampling pulses as shown in Fig. 4 were distorted. However, since the sampling points can be achieved by an integration of the sinc pulse sequences over its repetition time, this does not influence the measurement. Following the theory section, a photodiode and electronics bandwidth corresponding to the repetition time of the pulses (around 12 GHz) would be sufficient for the measurement.

**Figure 5:**
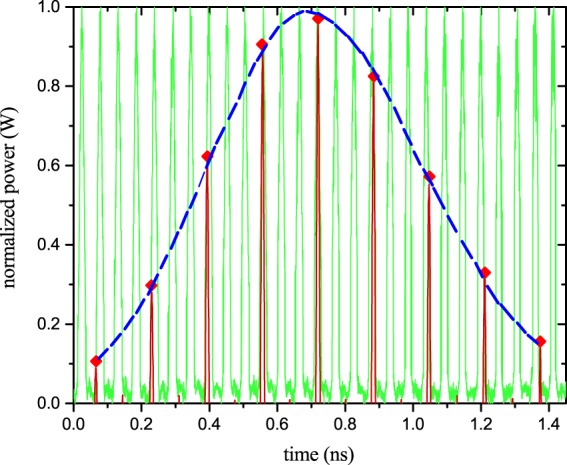
Stretching and sampling of the 15.55 ps input pulse (60 GHz). The signal copies at the output of the ring resonator (green) are multiplied with a sinc pulse sequence (red) by convolution in the frequency domain with a nine-line frequency comb with Δ*f*_*s*_ = 12.23 GHz. For better visualisation, the pedestal was removed, the output copies were normalized to the one with the highest amplitude and the sampling pulses were multiplied with the corresponding normalization factor. Since the ring behaves equally for all input signals, this can be done by post-processing of the data. The sampling points (red diamonds) are retrieved by an integration of the sampling pulses over their repetition time. Due to limitations of the PLL, the sampling frequency was not close to the repetition rate of the replicas Δ*f*_*R*_ = 18.774 GHz. Consequently, only every third copy could be sampled and every second sampling pulse is in the gap between two copies (small red lines). The stretched pulse is the envelope over the sampling points and has a stretching factor of Δ*S* = 44.

Since single pulses can be measured by the method, just one single phase adjustment is sufficient. As the time interval between the copies is fixed by the round-trip time, or the FSR of the ring, the sampling interval can be chosen such that each copy is sampled at a specific position. As described with Eq. (9) and shown in Fig. 5, this phenomenon can be exploited to stretch the input pulse in time. As the FSR of the ring was measured to be $\Delta {f}_{R}=18.774$ GHz, the temporal signal copies had a fixed time difference of around 53.265 ps. The sampling interval was chosen in a way that for each shot the sinc pulse sequence is multiplied with the copies at successive positions. Due to limitations of the PLL, the sampling frequency $\Delta {f}_{S}$ could not be adjusted close to the FSR of the ring. Hence, only every third copy has been sampled by the sinc pulse sequence. The result for a 60 GHz input pulse and a sampling frequency of $\Delta {f}_{S}=12.23$GHz can be seen in Fig. 5. Due to the difference between the repetition and the sampling frequency, the single pulse was stretched in time. Since only every third copy was hit by a sampling pulse, every second sampling pulse falls into the gap between two copies (see the small red lines in Fig. 5). Therefore, the effective sampling time is doubled, leading to an effective sampling frequency of $\Delta {f}_{Seff}=6.115$ GHz.

From Eq. (9) and with *m* = 3 (every third copy is sampled), the stretching factor can be calculated to be $\Delta S=44$ and the compressed sampling rate or frequency is $\Delta {f}_{CS}=\Delta {f}_{seff}\Delta S\approx 270$GSa/s. However, the effective compressed sampling rate depends on the bandwidth of the pulses and is $\Delta {f}_{CSeff}=N\Delta {f}_{S}\approx 110$ GSa/s, as will be discussed in the next section.

**Figure 6:**
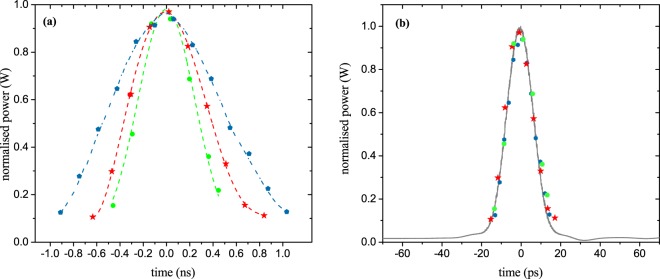
Stretching and sampling of the 15.55 ps input pulse with different sampling frequencies and resulting stretching factors. (**a**) By a small change of the sampling frequency Δ*f*_*S*_ from 12.15 GHz (green) via 12.23 GHz (red) to 12.34 GHz (cyan) the stretching factor Δ*S* can be altered from 34 via 44 to 71. Please note that here again, the effective sampling frequency is Δ*f*_*Seff*_ = Δ*f*_*S*_/2. In (**b**) the measured results of (**a**) are compressed by the corresponding stretching factor Δ*S*. The stars and dots correspond to (**a**). The black trace is the measured input pulse as already presented in Fig. 3(a). Evidently, the compressed pulses follow the original measurement very well.

By altering the sampling frequency, the stretching factor can be changed as reported in Fig. 6a. Here just three slightly different sampling frequencies have been used to achieve stretching factors of 34 (green dots), 44 (red stars) and 71 (cyan dots). If the measured data are compressed by $\Delta S$, they give the original input pulse, as shown in Fig. 6b. The black trace is the measured 15.55 ps input pulse (Fig. 3a) and the stars and dots correspond to the sampling points in Fig. 6a. As can be seen, the compressed measured data follow the input pulse very well. Therefore, we assume that dispersion in the ring or the setup does not lead to an inaccuracy in the measurement.

**Figure 7:**
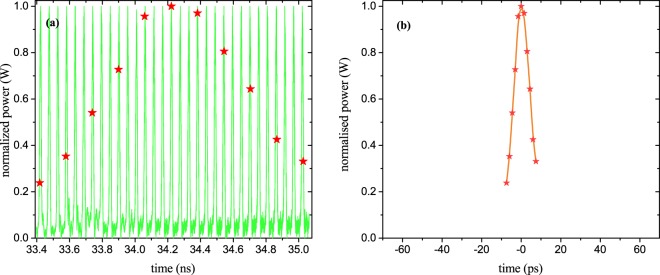
Sampled and stretched input pulse with a bandwidth of 100 GHz. (**a**) Post processed pulse copies (green) with a repetition rate of Δ*f*_*R*_ = 18.774 GHz sampled with an effective sampling frequency of 6.2 GHz (Δ*f*_*S*_ = 12.4 GHz, blue dots). The stretching factor is Δ*S* = 108. (**b**) Reconstructed input pulse with an FWHM duration of 10.37 ps.

Figure 7a shows the sampling of an input pulse with an optical bandwidth of 100 GHz defined by the waveshaper. Since this is at the bandwidth edge of the used photodiode, for the electronic measurement the pulse copies, as well as the sampling pulses, were distorted. However, since the sampling points are the integral of the sinc pulse sequence over its repetition time, this distortion does not influence the measurement with our method. The sampling points still give the time-stretched input pulse with a stretching factor of $\Delta S=108$. In Fig. 7b the input pulse was reconstructed from the sampled data. The FWHM duration of the reconstructed signal follows with 10.37 ps very well the expectations.

## Discussion

For the 100 GHz pulse, the compressed sampling rate is $\Delta {f}_{CS}=\Delta {f}_{seff}\Delta S\approx 670$ GSa/s. However, since the sinc pulses used for sampling have just about 1/6^th^ of this bandwidth, for such a sampling rate they cannot provide an independent information about the sampling points. Each bandwidth-limited signal can be written as a superposition of time shifted sinc functions:

10 $$s(t)=\mathop{\sum }\limits_{k=-\infty }^{\infty }s(\frac{k}{{B}_{{\rm{o}}}}){\rm{sinc}}({B}_{{\rm{o}}}t-k)$$

The bandwidth of this signal in the optical domain is *B*_o_ and correspondingly its baseband width *B*_o_/2. Since sinc functions are orthogonal, it can be shown that:

11 $${\int }_{-\infty }^{\infty }s(\frac{k}{{B}_{{\rm{o}}}}){\rm{sinc}}({B}_{{\rm{o}}}t-k)\times \mathrm{sinc}(N\Delta {f}_{S}t-k)dt=\frac{1}{{B}_{{\rm{o}}}}s(\frac{k}{{B}_{{\rm{o}}}})$$

and

12 $${\int }_{-\infty }^{\infty }s(\frac{k}{{B}_{{\rm{o}}}}){\rm{sinc}}({B}_{{\rm{o}}}t-k)\times \mathrm{sinc}(N\Delta {f}_{S}t-l)dt=0$$

with *k*, *l* ∈ *I*, *k* ≠ *l*, and *I* as the set of integer numbers. However, this only holds for sinc pulses with a bandwidth of $B=N\Delta {f}_{S}\ge {B}_{o}$. Therefore, to retrieve a distinct sampling value $s(\frac{l}{{B}_{o}})$, the signal has to be multiplied with a sinc pulse with a bandwidth of at least $B={B}_{o}$ to get:

13 $${\int }_{-\infty }^{\infty }\mathop{\sum }\limits_{k=-\infty }^{\infty }s(\frac{k}{{B}_{{\rm{o}}}}){\rm{sinc}}({B}_{{\rm{o}}}t-k)\times {\rm{sinc}}({B}_{{\rm{o}}}t-l){\rm{d}}t=\frac{1}{{B}_{{\rm{o}}}}s(\frac{l}{{B}_{{\rm{o}}}})$$

with $1/{B}_{{\rm{o}}}$ as a constant, which is not relevant for the concept. So, as long as the repetition rate is higher than the bandwidth of the sampling pulses, the maximum optical bandwidth of the signal which can be sampled is defined by the bandwidth of the sampling pulses and not by their repetition rate. Since the bandwidth of the sampling pulses used to sample the 100 GHz pulses was around 112 GHz, the effective compressed sampling rate is $\Delta {f}_{CSeff}=112\,{\rm{GSa}}/{\rm{s}}$ and the maximum optical bandwidth that can be sampled with these pulses is below 112 GHz. Thus, the corresponding width in the baseband will be below 56 GHz.

The pulse bandwidth and therefore the effective compressed sampling rate is defined by the RF bandwidth of the used modulators. With two cascaded modulators the maximum achievable pulse bandwidth for a frequency comb with an even number of lines is four times and for an odd number, three times their RF bandwidth^31^. Since integrated modulators with a bandwidth of 100 GHz have been shown^34,38^, this would correspond to an integrated sampling with sampling rates of up to 400 GSa/s and baseband bandwidths of 200 GHz.

It can be shown that the presented convolution in the two cascaded modulators can be used to retrieve the phase of the sampling point by comparison with the phase of a local oscillator^33^. So if instead of a simple photodiode a coherent receiver will be used to integrate the sampling points, a full-field sampling can be accomplished. Hence, much higher bandwidth signals can be measured by a combination of the method with spectral slicing^8^. Compared to conventional spectrum slicing methods^8^, the method requires much lower bandwidth electronics and reduces the number of parallel channels. Accordingly, by electronics and photonics co-integration, fully integrated, ultra-high bandwidth measurement devices would be possible on a silicon-on-insulator platform.

Another important parameter defining the resolution of the measurement is the jitter. For the method presented here, two important jitter contributions can be distinguished. The first is the ring itself and the other is the RF source. Temperature changes of the ring can be compensated by an active temperature control in order to keep the free spectral range and therefore the repetition rate of the time copies constant. However, another possibility would be to monitor the change of the FSR by a PD, as described below, and to consider it in the post processing. The RF source driving the two cascaded modulators can be replaced by sources with ultra-low jitter. These sources can reach jitter values in the zeptosecond range^39^ and show a phase noise down to −167 dBc/Hz at an offset of 10 kHz^40^.

For the proof-of-concept experiment, the two cascaded modulators were synchronized with the source. However, for the magnification of an isolated single event, the synchronization of the repetition rate of the signal replicas with the sampling interval is crucial. This can be achieved by a PD with a bandwidth of the rings FSR at the through port of the ring. This PD would not only monitor the FSR of the ring but, as well the power distribution between individual copies for post processing.

In the proof-of-concept setup, use of optical amplifiers was necessary to compensate coupling losses. A fully integrated system comprising the ring, the coupled modulators, and a photodiode may avoid an amplification. Due to high Q resonance of the ring, it still might have high losses. Nevertheless, a variable bus waveguide to ring coupling can solve this issue.

In conclusion, we have presented a new optical time magnification method for the sampling of fast single events, which neither require any dispersion nor nonlinearities. The proof-of-concept experiments were carried out with an integrated silicon microring resonator and commercial Lithium Niobate intensity modulators. However, an integration of the whole setup on a CMOS platform might be straightforward. The method does not only stretch the input signal but at the same time, it samples the shape of the signal. With 40 GHz modulators, we have shown the sampling of signals with an optical bandwidth of up to 100 GHz and an effective compressed sampling rate of around 112 GSa/s. Except for the frequency tripler, electronic equipment and a photodiode with a maximum bandwidth of 12 GHz would have been sufficient. The achieved stretching factor was up to 108. The measurement bandwidth is restricted by the RF bandwidth of the used modulators. With integrated 100 GHz modulators^34,38^, integrated devices with effective compressed sampling rates of 400 GSa/s are possible. Since the whole field (amplitude and phase) can be sampled^33^, much higher measurement bandwidths can be achieved by spectrum slicing^8^. Since bandwidths of 100 GHz can be characterized with standard silicon photonic CMOS compatible devices and low bandwidth electronics (12 GHz), a 100 GHz wavelength division multiplexer combined with the described method, spectrum slicing and co-integrated electronics could enable the single shot measurement of very short events with THz bandwidths in integrated, low bandwidth CMOS compatible devices.

## Methods

### Experimental setup

**Figure 8:**
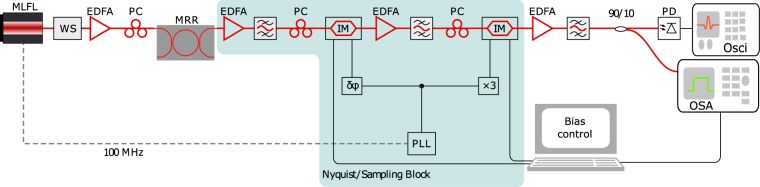
Experimental Setup. The pulses are generated by a mode-locked fiber laser (MLFL), restricted in bandwidth by a waveshaper (WS) and fed into the microring resonator (MRR). The temporal characterization will be done by an electrical sampling scope (Osci), whereas the spectral characterization will be carried out by an optical spectrum analyzer (OSA). Two cascaded intensity modulators with synchronized frequency inputs serve as the Nyquist block, which samples the signal. PC: polarization controller, IM: Lithium Niobate Mach-Zehnder intensity modulator, EDFA: erbium doped fiber amplifier, PLL: phase locked loop for RF signal generation, PD: photodiode.

The experimental setup is shown in Fig. 8. The used laser source was a passively mode-locked femtosecond Er-doped fiber laser (FemtoFErb 1560). The overall bandwidth and the repetition rate of this laser was 30 THz and 100 MHz respectively. In order to be measurable by available instruments, the bandwidth of the input signal was reduced to 60 GHz or 100 GHz by a waveshaper. Before feeding to the microring resonator (MRR), the output of the waveshaper was amplified by an Erbium doped fiber amplifier (EDFA) to 12 dBm of average power. The light was coupled into the MRR by using inverted tapers on the integrated waveguides and lensed tapered single-mode fibers. The insertion loss of the fiber-to-chip coupling was estimated to be around 6 dB, based on the measurements on a straight waveguide. The input polarization was suitably adjusted using a fiber polarization controller with an insertion loss of around 1 dB. Therefore, the optical power in the input bus waveguide of the ring was around 5 dBm. Due to the coupling losses of the ring, the optical power in the ring was much lower. As the spectral bandwidth (60 GHz, or 100 GHz) of the input signal is much larger than the free spectral range of the microring resonator (18.774 GHz), the pulse initially couples into the resonator and travels around the ring. At each pass, it couples energy out to the two adjacent waveguides. This results in a series of output pulses with a diminishing amplitude at the drop port. The repetition rate of the input pulse (100 MHz) was so low, that there was no optical power left in the resonator before the next input pulse arrived. The chip was placed on a temperature stabilized stage with stabilization of 27 ± 0.001 °C.

The generated pulse copies were then subjected to the all-optical sampling stage (Nyquist/sampling block in Fig. 8), which consists of two cascaded Lithium Niobate Mach-Zehnder modulators (MZM). The MZMs were driven by a sinusoidal RF signal generated by a PLL which uses the repetition rate of the femtosecond laser as the reference signal for the phase locked operation. The second MZM was driven by a frequency which was three times higher than the driving frequency of the first MZM. A passive frequency tripler was used for this purpose. An RF phase shifter was used to compensate any phase mismatch between the RFs at the input of the two MZMs. The position of the sampling points can be arbitrarily controlled by changing the phase of the RF frequency generated by the PLL. For the sampling stabilization, the MZM bias points were controlled suitably to get a nearly flat top spectrum at the OSA. For the proof-of-concept experiment, the PLL was necessary for the synchronization of the input sampling frequency to the signal to sample. For single, non-periodical events, this can be achieved by synchronizing the sampling frequency to the ring repetition rate.

All the time domain measurements were carried out with a 100 GSa/s photodiode (Finisar XPDV412xR) along with a sampling oscilloscope (Agilent DCA-J 86100C) equipped with an additional measurement head of 70 GHz bandwidth (Agilent 86118A). Due to this limitation in measurement, the measured sampling pulses are distorted although the integration over one period will still give the correct sampling value. Thus, just for getting the sampling points a photodiode and measurement equipment with 12 GHz bandwidth would have been sufficient. The original pulse as well as the replicas at the output of the ring were measured with the same measurement devices.

### Design and fabrication of the nitride microring resonator

We designed a group of silicon nitride ring resonators with different sizes. The silicon nitride waveguides are 1.8 μm wide and 800 nm high. The waveguide sidewall angle is 82° due to the dry etch. The device that we measured in Fig. 2 (ring 1) is a racetrack-type ring resonator with the straight section being 3462 μm long and bending radius 100 μm. The ring resonator is coupled with input and output access waveguides via S-bends at the long straight sections. The S-bend has a bending radius of 100 μm and a gap separation of 0.4 μm from the ring resonator. The fabrication was done in LIGENTEC. A 500-μm-thick silicon wafer was initially oxidized to form a 4-μm-thick silicon dioxide layer as the under-cladding of the device. Then the 800-nm-thick core silicon nitride layer was deposited using low-pressure chemical vapor deposition (LPCVD). The strip waveguides are patterned with electron-beam lithography and transferred to the substrate by reactive ion etching (RIE) with SF_6_/CH_4_ chemistry. Finally, the device was covered by 2.7-μm-thick silicon dioxide as upper-cladding deposited by plasma-enhanced chemical vapor deposition (PECVD). The accuracy in the layer thickness is 1% in the thickness of the deposited layers.
